# Can ChatGPT Fool the Match? Artificial Intelligence Personal Statements for Plastic Surgery Residency Applications: A Comparative Study

**DOI:** 10.1177/22925503241264832

**Published:** 2024-07-23

**Authors:** Jeffrey Chen, Brendan K. Tao, Shihyun Park, Esta Bovill

**Affiliations:** 1 Michael G. DeGroote School of Medicine, 62703 Faculty of Health Sciences, McMaster University, Hamilton, Ontario, Canada; 2 12358 Faculty of Medicine, University of British Columbia, Vancouver, British Columbia, Canada; 3 70382 School of Pharmacy, University of Waterloo, Kitchener, Ontario, Canada; 4 Division of Plastic Surgery, University of British Columbia, Vancouver, British Columbia, Canada

**Keywords:** residency, artificial intelligence, ChatGPT, machine learning, postgraduate medical education, apprentissage machine, ChatGPT, éducation médicale postdoctorale, intelligence artificielle, résidence

## Abstract

**Introduction:** Personal statements can be decisive in Canadian residency applications. With the rise in AI technology, ethical concerns regarding authenticity and originality become more pressing. This study explores the capability of ChatGPT in producing personal statements for plastic surgery residency that match the quality of statements written by successful applicants. **Methods: **ChatGPT was utilized to generate a cohort of personal statements for CaRMS (Canadian Residency Matching Service) to compare with previously successful Plastic Surgery applications. Each AI-generated and human-written statement was randomized and anonymized prior to assessment. Two retired members of the plastic surgery residency selection committee from the University of British Columbia, evaluated these on a 0 to 10 scale and provided a binary response judging whether each statement was AI or human written. Statistical analysis included Welch 2-sample *t* tests and Cohen's Kappa for agreement. **Results:** Twenty-two personal statements (11 AI-generated by ChatGPT and 11 human-written) were evaluated. The overall mean scores were 7.48 (SD 0.932) and 7.68 (SD 0.716), respectively, with no significant difference between AI and human groups (*P* = .4129). The average accuracy in distinguishing between human and AI letters was 65.9%. The Cohen's Kappa value was 0.374. **Conclusions:** ChatGPT can generate personal statements for plastic surgery residency applications with quality indistinguishable from human-written counterparts, as evidenced by the lack of significant scoring difference and moderate accuracy in discrimination by experienced surgeons. These findings highlight the evolving role of AI and the need for updated evaluative criteria or guidelines in the residency application process.

## Résumé

**Introduction:** Les lettres personnelles peuvent être décisives lors des candidatures canadiennes à la résidence. Étant donné l’intérêt pour la technologie d’IA, les préoccupations éthiques au sujet de l’authenticité et de l’originalité deviennent plus urgentes. La présente étude vise à établir si ChatGPT peut produire des lettres personnelles pour la résidence en chirurgie plastique qui correspondent à la qualité de celles rédigées par des candidats retenus. **Méthodologie:** ChatGPT a été utilisé pour produire une cohorte de lettres personnelles destinées au CaRMS (Service canadien de jumelage des résidents) afin de les comparer à celles de candidatures en chirurgie plastique retenues par le passé. Chaque lettre produite par l’IA et rédigée par un humain a été randomisée et anonymisée avant l’évaluation. Deux membres retraités du comité de sélection à la résidence chirurgie plastique de l’Université de la Colombie-Britannique ont évalué les lettres sur une échelle de 0 à 10 et ont donné une réponse binaire pour établir si chacune était produite par l’IA ou rédigée par un humain. L’analyse statistique incluait des tests à deux échantillons de Welch et le coefficient Kappa de Cohen pour en vérifier la concordance. **Résultats:** Les évaluateurs ont examiné 22 lettres personnelles (11 produites par IA—ChatGPT—et 11 rédigées par un humain). Les scores moyens globaux s’élevaient à 7,48 (ÉT 0932) et 7,68 (ÉT 0716), respectivement, sans différence importante entre le groupe d’IA et le groupe humain (p = 0,4129). L’exactitude moyenne à distinguer les lettres rédigées par des humains de celles produites par l’IA atteignait 65,9%. La valeur Kappa de Cohen était de 0374. **Conclusions:** ChatGPT peut produire des lettres personnelles pour les candidatures à la résidence en chirurgie plastique, d’une qualité indissociable de celles rédigées par des humains, comme le démontre le peu de différence entre les scores et l’exactitude modérée de la discrimination par des chirurgiens d’expérience. Ces observations démontrent le rôle évolutif de l’IA et la nécessité de mettre à jour les critères ou les directives d’évaluation lors du processus de demande en résidence.

## Introduction

The Canadian Resident Matching Service (CaRMS) serves as the national annual residency selection process in Canada, offering medical students a centralized platform to apply for postgraduate training programs.^1^ This process involves the submission of a CaRMS application package, followed by a thorough file review by the programs, and ultimately, candidate interviews.^1^

Personal statements have traditionally held to be potentially decisive in residency applications, as relying solely on objective data for selection has been shown to be both an inadequate predictor of clinical performance and leave gaps in a candidate's full portrait.^2,3^ These letters offer insights into applicants’ qualities, experiences, and motivations beyond their academic achievements.^1^ A recent survey study revealed that Canadian program directors of Plastic Surgery residency programs value personal statements with a rating of 3.1 on a 5-point Likert scale, equating their utility to that of medical school transcripts.^4^ Similarly, program directors and selection committee members of Canadian urology residency programs rated the importance of the personal statement at 3.65 on the same scale.^5^ In the United States, between 74% and 78% of program directors consider personal statements important in selecting interview candidates, and 48% to 54% rely on them when determining final rank lists.^3,6^ Studies have further demonstrated a correlation between applicant traits found in the personal statement and decreased resident attrition.^7,8^ Nonetheless, despite their utility in candidate selection, personal statement scoring remains highly subjective, resulting in poor interrater reliability as there is often a lack of objective criteria.^9,10^

Residency applicants invest substantial time in crafting and refining their personal statements, leading to a significant level of anxiety in 80% of these applicants.^11^ Recently, advanced AI language models, such as ChatGPT (OpenAI, San Francisco), have shown potential in automating the generation of personalized and persuasive narratives, including personal statements, given its high accuracy operating natural language generation and ability to write essays of reasonable quality on a particular topic.^12^ Other evidence demonstrates the utility of ChatGPT in writing patient medical educational handouts with acceptable quality.^13^ However, outside the context of medical writing, it is important to consider the limitations of ChatGPT in other writing circumstances, such as lack of context or personalization, ethical concerns, and inaccurate information.^12^ To address these issues, human experts may provide guidance, context, and verification to enhance ChatGPT's output.^12^ Nonetheless, a controversial study revealed that human reviewers accurately identified only 68% of ChatGPT-generated artificial research paper abstracts.^14^ Whether these limitations extend to the writing of reference letters for residency applications remain underinvestigated.

This study aims to explore the capability of AI in producing convincing personal statements for plastic surgery residency that match the quality of statements written by successful applicants. If ChatGPT-generated statements prove to be equally persuasive and impactful, it prompts the question of how much weight should be placed on personal statements in the selection process today. Understanding the potential of AI in assisting with personal statement writing can inform selection committees across Canada of the need for a more balanced and fair selection criteria, particularly in an era of machine learning. The primary outcome of this study is the subjective score of each group evaluated by 2 plastic surgery faculty from the University of British Columbia, experienced in CaRMS selection. The secondary outcome is a binary question to assess if the plastic surgeon evaluators can differentiate whether the personal statement is written by an applicant or ChatGPT.

## Methods

### Study Design

The aim of this study is to assess the ability of ChatGPT 4.0 in generating personal statements for plastic surgery residency applications, simulating the circumstances of a medical student. Chat-GPT was selected for its open access and equipped with universally accessible online resources to facilitate this simulation. A guide published in the *Journal of Graduate Medical Education*, authored by clerkship and residency program directors with a combined experience of 50 years, has underscored the role of personal statements in highlighting an applicant's strengths and achievements.^3^ This guide further suggests incorporating personal anecdotes and patient stories as a means to showcase personal traits, the journey toward or enthusiasm for the chosen discipline, and professional growth.^3^ These elements, along with a structured framework, were woven into the ChatGPT prompt to ensure sufficient variety.^3^

ChatGPT-4 was utilized using the following query stem on November 18, 2023:

I am a final year medical student in Canada applying for a plastic surgery resident program at the University of British Columbia. Please write me a personal statement with no fixed structure of a maximum of 500 words incorporating specific and compelling stories, experiences, or something that introduces the applicant and makes the reader want to know more, and essential details that a program must know about the applicant and their proudest accomplishments, and specific strengths related to the specialty of choice and leadership experiences. Please do not use placeholders.

To avoid any potential influence from prior inputs, each query was executed in a fresh ChatGPT session. Furthermore, to account for response quality variation, we repeated this search eleven times, evaluating and rating each answer individually. All identifiable information has been removed and replaced with variables.

Human-written personal statements submitted to CaRMS from 2018 to 2023 were collected, with permission, from current UBC plastic surgery residents by EB and anonymized prior to assessment.

### Data Collection and Grading

Each ChatGPT-generated and human-written statement was assessed blindly by 2 plastic surgeons, recently retired from the UBC CaRMS selection committee. In total, 11 successful statements were compared with 11 ChatGPT-generated statements evaluated on a subjective score range (0-10), replicating the residency application process at University of British Columbia. The secondary outcome, binary response discriminating past successful versus AI-generated personal statements were collected in the same manner.

### Statistical Analysis

The primary outcome was the surgeons’ rating score (scale: 0-10) between human- and AI-derived reference letters. We refer to the surgeons as “Surgeon A” and “Surgeon B.” The secondary outcome was the likelihood of correct prediction of human- versus AI-derived reference letters. Descriptive statistics were described as mean (standard deviation; SD) or median (interquartile range; IQR), where applicable. Inferential statistics were conducted using independent pairwise (Welch 2-sample) *t* test to determine significant between-group differences, using an a = 0.05. For each surgeon, we calculate their sensitivity, specificity, and accuracy of detecting human-from-AI-written letters. We employ Cohen's Kappa analysis to determine agreement between the surgeon's rating of human versus AI. Agreement on continuous quality scoring was assessed using a Bland-Altman plot. Where indicated, correlation analysis was conducted using Spearman methodology. Analysis was completed by BT in RStudio (version 2023.06.1 + 524).

### Ethical Considerations

This study adheres to the principles of the Declaration of Helsinki. In accordance with article 2.4 of the tricouncil policy statement for research ethics in Canada, institutional review board approval was not required as the data were obtained from publicly available sources. No personally identifiable information was generated or used in this study.

## Results

Across 22 personal statements (11 from ChatGPT and 11 human-written), Surgeons A and B rated an overall mean (SD) score of 7.48 (0.932) and 7.68 (0.716), respectively. Table 1 depicts both surgeons’ scoring when stratified by human- versus AI-derived letters. When both raters’ scores were pooled into a summary estimate, there was no significant difference in scoring between AI and human groups (*P* = .4129).

**Table 1.** Surgeons’ Scoring of Human- Versus AI-Derived Letters.

	Human-derived letters (mean, SD)	AI-derived letters (mean, SD)
Surgeon A	7.5 (0.866)	7.45 (1.04)
Surgeon B	7.91 (0.701)	7.45 (0.688)
Pooled Scoring	7.70 (0.697)	7.45 (0.705)

**Figure 1.**
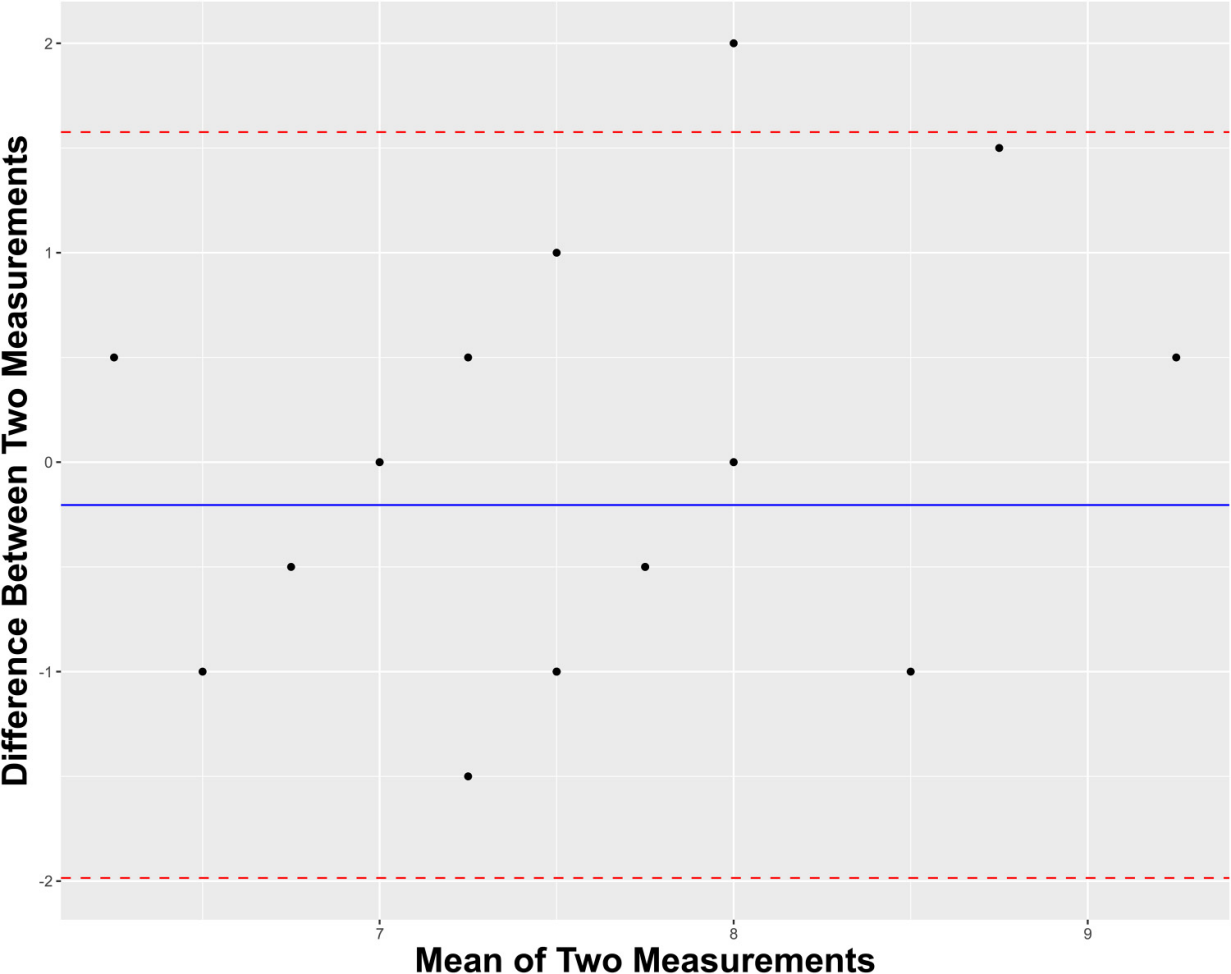
Bland-Altman plot for intersurgeon agreement on scoring letters.

With respect to discriminating between human- and AI-derived letters, Surgeon A exhibited a sensitivity, specificity, and accuracy of 0.636, 0.818, and 0.727, respectively. Comparatively, Surgeon B exhibited a sensitivity, specificity, and accuracy of 0.636, 0.545, and 0.591, respectively. Regarding interrater reliability of discerning human-written material from not, Surgeons A and B shared a Cohen's Kappa of 0.374, indicating fair agreement. Figure 1 depicts a Band-Altman plot to visualize the intersurgeon agreement on their continuous variable scoring of each letter. The overall mean difference of scoring between raters was near-zero with all-but-one individual mean differences lying within the 95% confidence interval of the pooled mean difference, thereby suggesting acceptable agreement.

## Discussion

This study suggests AI is capable of generating convincing, high-quality personal statements for Plastic Surgery residency applications with minimal guidance. Our results contribute new and objective data to the timely discussion around the complex ethical challenge of distinguishing between AI-written and human-written academic pieces.^15^ Our comparative analysis revealed no significant difference in average ratings between AI and human groups. This observation is found between unaltered AI without any post-generation editing, and human-written statements, and may even underestimate the potential strength of a hybrid AI-human approach, such as sequential refinement of query stems. This study adds to the question of AI in enhancing research and academic productivity, and its impact on the traditional functions of academic institutions with the possible blurring of lines between human creativity and machine efficiency.^16^

The ethical and practical implications of AI use in academia are profound and multifaceted. The utilization of AI in academic pursuits, especially in contexts such as lesser-known journals, graduate theses, and grant applications, brings forth issues of authenticity and academic integrity.^17^ A notable example, the admission of a Japanese literature prize winner who utilized ChatGPT, illustrates the complex interplay of AI in creative and academic fields.^18^ This underscores the need for clear guidelines and best practices in AI usage. The CaRMS Privacy Statement emphasizes the responsibility of applicants to ensure the accuracy and completeness of their personal information.^19^ However, there is a notable absence of specific directives regarding AI assistance in this context. To mitigate potential issues such as the homogenization of statements, perpetuation of bias, and ethical dilemmas, it is imperative to define the role of AI in residency applications more clearly before establishing such guidelines.^20^ While current AI-detection tools (OpenAI, CrossPlag, GPTZero, and more) offer some capability to distinguish between human and AI-generated content, their performance is inconsistent, leading to the suggestion that they should not be the sole determinant in questions of academic integrity.^21^ Unlike plagiarism detection tools in widespread use throughout academia, AI screening may not yet be sufficiently robust to ensure a fair judgment. Thus, a broader, more nuanced approach is required to address the challenges posed by AI in academic settings.

The demonstrated success of AI in crafting personal statements of course risks diminishing their value, potentially shifting emphasis toward more quantifiable metrics like academic performance.^20^ CaRMS does not yet afford a standardized academic selection tool; the inevitable disparity between Medical School evaluation standards, together with the universally high grades presented by Plastic Surgery aspirants, confers inherent inadequacy on the ability to discriminate between them. Furthermore, the recent shift in Canadian medical education toward pass-fail and practical clinical evaluations, with less emphasis on competition and academic ranking, complicates the selection process.^4^ In highly competitive specialties, application volume exceeds the ability to meaningfully interview all candidates. Academic performance alone may correlate with postsecondary success but is limited in addressing suitability for a surgical career. References are highly subjective and often based on but momentary exposure.^22^ Personal statements offer a holistic view of the applicant's unique experiences and journeys that are not objectively measurable, and have historically therefore, formed an integral part of the residency application.^23^ Yet, the results from our study call for transparency about how programs will evaluate applications in this new AI-influenced landscape.^24^

As AI technology advances and its capability to generate increasingly sophisticated personal statements grows, ethical concerns regarding authenticity and originality become more pressing. The debate about the future role and relevance of personal statements in the context of emerging AI technologies is ongoing. As we are unlikely to stop the tide, will traditional personal statements become obsolete? Optimistically, a timely published guideline warning against the overt or inappropriate use of AI in CaRMS applications or policies regarding AI usage published by individual programs in their program descriptions may be sufficient. Additionally, publication of this and similar studies alerts applicants to selection committee self-awareness. This allows for bilateral acceptance of AI as a valid academic tool within ethical boundaries. The determined student will exploit AI while honing its contribution with a personal slant. Meanwhile, the shrewd reviewer will become more discerning against generic content and AI flags, rewarding effort invested in authenticity and originality. Nevertheless, our study implies that AI will probably outpace the evolving capability of human subjectivity, and ongoing prospective validation of *all* application tools should be promoted to preserve their discriminatory value.

### Strengths and Limitations

The limitations of this study include its small sample size, which may not represent a broader range of personal statements. The subjective nature of the evaluation process and potential biases is also concerns, especially considering that our faculty, while chosen for their retired status, may not fully capture the range of experiences of a current file reviewer, particularly in terms of familiarity with AI. Nonetheless, the reviewers’ more than 20 years of experience involved in CaRMS selection process remain a notable strength of this study. Future works could therefore iterate upon this work by evaluating whether these results generalize well across a greater number of raters and further explore the implications of AI in academic settings.
